# A germline *FLT3* variant in aplastic anemia

**DOI:** 10.1186/s40364-024-00717-3

**Published:** 2025-01-07

**Authors:** Lemchukwu C. Amaeshi, Amalia A. Sofianidi, Aditi Shastri, Mendel Goldfinger, Marina Konopleva, Amit K. Verma, Mark Chaitowitz, Ioannis Mantzaris

**Affiliations:** 1 Montefiore Medical Center, Bronx, NY USA; 2 Albert Einstein College of Medicine/Montefiore Medical Center, Bronx, NY USA; 3 Department of Biological Chemistry, Medical School, National and Kapodistrian University of Athens, Athens, Greece; 4 Department of Oncology, Montefiore Medical Center and Albert Einstein College of Medicine, Bronx, NY USA; 5 Department of Leukemia, The University of Texas MD Anderson Cancer Center, Houston, TX USA

**Keywords:** Aplastic anemia, *FLT3* variant, Germline variant, Pancytopenia, Autoimmunity, Dendritic cells

## Abstract

*FMS-like tyrosine kinase 3* (*FLT3*) genetic variants are commonly seen in high-grade myeloid neoplasms and are typically gain-of-function mutations associated with a proliferative disease phenotype. Inactivating *FLT3* variants have been less frequently described in non-malignant, autoimmune disorders and are uncommon in aplastic anemia (AA). Herein, we report the first to our knowledge, and unusual case of a germline, gain-of-function, *FLT3* variant in a patient with severe AA treated successfully with immunosuppressive therapy. Although a proposed link between dysregulated *FLT3* signaling and autoimmunity has been described and could be speculated in the case of AA, it is currently unknown whether a pathogenetic connection between an activating germline *FLT3* variant and AA truly exists and whether the mutation signifies a lifelong risk of disease recurrence and/or clonal evolution. However, the recognition of the *FLT3* gene as subject not only to somatic but also germline mutations is the first step in interrogating its functional implications. Further study of unusual genotype-phenotype combinations, such as in the case presented, may shed light on a potential pathogenetic link.

**Supplementary Information:**

The online version contains supplementary material available at 10.1186/s40364-024-00717-3.

To the editor,

We present an unusual case of severe aplastic anemia (SAA) in a patient harboring a gain-of-function, presumed-germline *FMS-like tyrosine kinase 3* (*FLT3*) variant.

## Case summary

A 57-year-old African American woman was incidentally found to have pancytopenia during a pre-operative workup (Table 1). Her complete blood count up to six years prior was notable for intermittent mild normocytic anemia and neutropenia. She reported a negative known family history of hematologic disorders. The initial evaluation was unrevealing (Table 2), except for a low-titer positive ANA screen without clinical evidence of active autoimmune disease. The workup for paroxysmal nocturnal hemoglobinuria (PNH) showed no evidence of a PNH clone in flow cytometric analysis. Bone marrow aspiration and biopsy revealed marrow hypocellularity (5–20%) without an increase in blasts, and the marrow karyotype was normal. Next-generation sequencing (NGS) revealed a gain-of-function *FLT3 c.2039 C > T* (p.Ala680Val) variant with an allele frequency (VAF) of 45.9%. No other variants were detected (NeoTYPE™ Myeloid Disorders Profile, NeoGenomics Laboratories, Inc.). The high VAF in the absence of the usual proliferative phenotype of a FLT3-mutated myeloid neoplasm raised suspicion of germline origin, which was further supported by the detection of the variant in both hematopoietic (blood) and presumably non-hematopoietic tissue (saliva) (Cairo Diagnostics, LLC). The patient received immunosuppressive therapy, and she achieved hematologic remission. A surveillance marrow biopsy one year later showed normal trilineage hematopoiesis (bone marrow cellularity of 20–50%) and persistence of the *FLT3 c.2039 C > T* (p.Ala680Val) variant with a VAF of 52.1%.

**Table 1:** Complete blood count parameters before and at the time of diagnosis of aplastic Anemia

Timeline/Parameters	Six years before presentation	Pre-treatment initiation	One-year post-treatment	Reference range
Hemoglobin (g/L)	117	76	120	122–153
Mean Cell Volume (fL)	90.9	95.3	104.5	80–96
WBC (x10^9^/L)	4.3	1.1	4.6	4.8–10.8
ANC (x10^9^ /L)	1.4	0.2	1.5	1.8–7.7
ALC (x10^9^/L)	2.3	0.9	2.6	1.0–4.8
Platelet count (x10^9^/L)	209	13	165	150–400
Absolute reticulocyte count (x10^9^/L)	N/A	40.50	143.0	38.4–114.8

WBC: White blood cell count; ANC: absolute neutrophil count; ALC: absolute lymphocyte count; fL: femtoliter; N/A: Not available

**Table 2:** Initial evaluation of pancytopenia

Parameter	Result	Reference range
Absolute reticulocyte count (cells/mL)	52.9	38.4–114.8
Vit B12 (pmol/L)	> 1100	147.0–738.0
Folate (nmol/L)	32.0	6.8–34.0
Iron (mmol/L)	21.7	11.6–31.3
Iron binding capacity (mg/dl)	380.0	250.0-410.0
Ferritin (mg/L)	148.6	10.0-150.0
Transferrin (g/L)	3.0	2.0-3.6
Hepatitis C virus antibody	Non-reactive	
Hepatitis B surface antigen	Non-reactive	
HIV test	Negative	
TSH (mIU/L)	0.9	0.3–4.2
Copper (mmol/L)	28.7	11.0-27.6
ANA screen	Positive	
ANA titer	1:40	< 1:40
ANCA-P3	< 0.2	
ANCA-MPO	< 0.2	
SPEP	Negative for M-protein	

TSH: thyroid-stimulating hormone; ANA: Anti-nuclear antibody; ANCA-P3: Anti-proteinase 3 antibody,;ANCA-MPO: Anti-myeloperoxidase antibody; SPEP: serum protein electrophoresis; M-protein: monoclonal protein

## Discussion

Aplastic anemia (AA) is a disease characterized by peripheral cytopenia and trilineage bone marrow aplasia, with a complex pathophysiology implicating immune dysregulation, telomere attrition, and clonal hematopoiesis and a lifelong risk of clonal evolution to a myeloid neoplasm [1, 2]. Somatic *FLT3* variants have not been reported in AA, but four patients with SAA harboring germline, inactivating *FLT*3 variants, were recently described [3]. This is the first report of a gain-of-function presumed-germline *FLT3* tyrosine kinase variant in a patient with SAA.

The *FLT3* gene encodes the FLT3 receptor found on hematopoietic stem cells (HSC) [4], which, when activated by the FLT3 ligand produced by bone marrow stroma cells, mediates downstream signaling to promote HSC survival, proliferation, and differentiation [5]. Variants of the *FLT3* gene are typically somatic events leading to constitutive FLT3 receptor activation and are predominantly seen in acute myeloid leukemia (AML) with a proliferative phenotype. The *FLT3 c.2039 C > T* (p.Ala680Val) has been previously described as one such activating variant, originating from a missense mutation on the kinase domain of the FLT3 receptor [6, 7], but its discovery in this patient’s case is unusual and noteworthy. First, our patient presented with marrow hypoplasia with no evidence of an abnormal cell population, and second, the *FLT3 c.2039 C > T* (p.Ala680Val) variant was seen in almost 50% of the cell DNA analyzed, suggesting germline origin, subsequently corroborated by its detection in presumably non-hematopoietic tissue.

The key question raised is whether, in fact, the *FLT3* genetic variant played a pathogenic role in this patient’s AA, and if so, whether this implies a lifelong risk of disease recurrence and/or clonal evolution above and beyond the risk associated with acquired AA, without germline variants. Outside hematologic malignancies, FLT3 signaling plays a role in dendritic cell (DC) development and function [8]. These antigen-presenting cells modulate T-cell immune responses and have the potential to initiate autoimmunity [9, 10]. In AA, reports of *FLT3* genetic variants are limited to a recent case series of 4 patients with germline inactivating mutations [3]. These patients subsequently acquired somatic variants in the other *FLT3* allele or genes encoding other phosphotyrosine receptor kinases. The authors hypothesized that a maladaptive somatic genetic rescue in response to the familial *FLT3* haploinsufficiency was the underlying mechanism leading to the somatic events. However, a causal relationship between the inactivating germline FLT3 variant and AA phenotype was not proposed. Similarly to what has been suggested in autoimmune diseases [11], loss-of-function *FLT3* variants and compensatory upregulation of the FLT3 ligand could lead to a dysregulated immune response manifesting as AA. It is also conceivable that a gain-of-function variant could promote DC dysregulation via constitutively active FLT3 signaling, which could trigger a T-cell-mediated attack on hematopoietic cells.

Whether there is a pathogenetic connection between the presumed germline *FLT3* variant and the development of severe AA or whether the germline variant poses a lifelong risk of disease recurrence or clonal evolution remains a matter of speculation. The unexpected finding of an activating germline *FLT3* variant highlights the need to further explore the functional implications of such germline variants in this gene. Careful detection of new cases should enable future studies of similarly unusual genotype-phenotype combinations to shed light on potential pathogenetic links and explore their therapeutic implications.

## Electronic supplementary material

Below is the link to the electronic supplementary material.

Supplementary Material 1

Supplementary Material 2

Supplementary Material 3

## Data Availability

No datasets were generated or analysed during the current study.

## Abbreviations

AA: Aplastic anemia
AML: Acute myeloid leukemia
DC: Dendritic Cells
HSC: Hematopoietic stem cells
FLT 3: FMS-like tyrosine kinase 3
MDS: Myelodysplastic syndrome
NGS: Next generation sequencing
VAF: Variable allele frequency
