# Supplementary figures and images for: A germline FLT3 variant in aplastic anemia
Source: Biomark Res. 2025 Jan 7;13:4. doi: 10.1186/s40364-024-00717-3 (PMC11707911; doi:10.1186/s40364-024-00717-3)

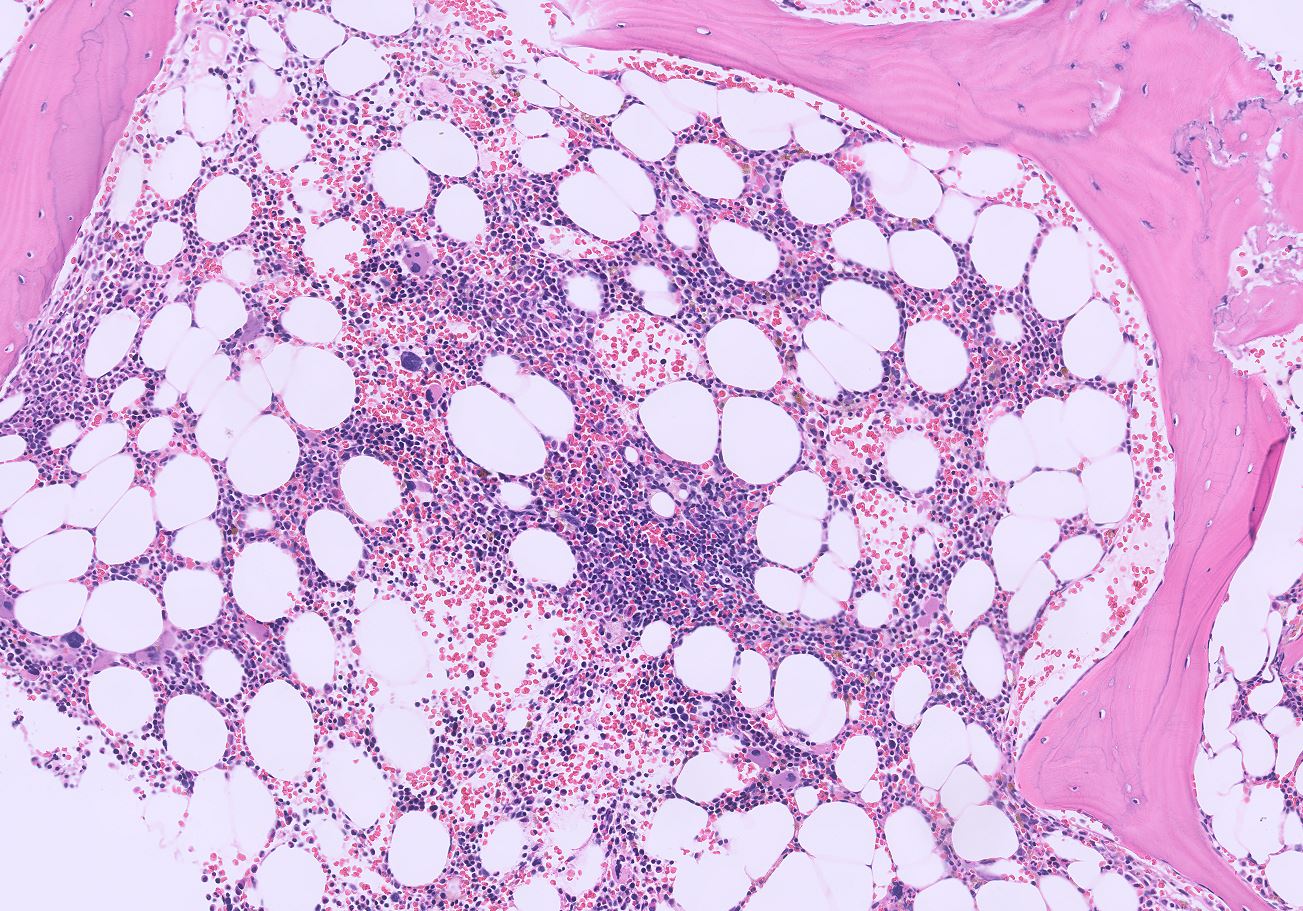

Supplement: Supplementary file 1 — Supplementary Material 1 [file 40364_2024_717_MOESM1_ESM.jpg]

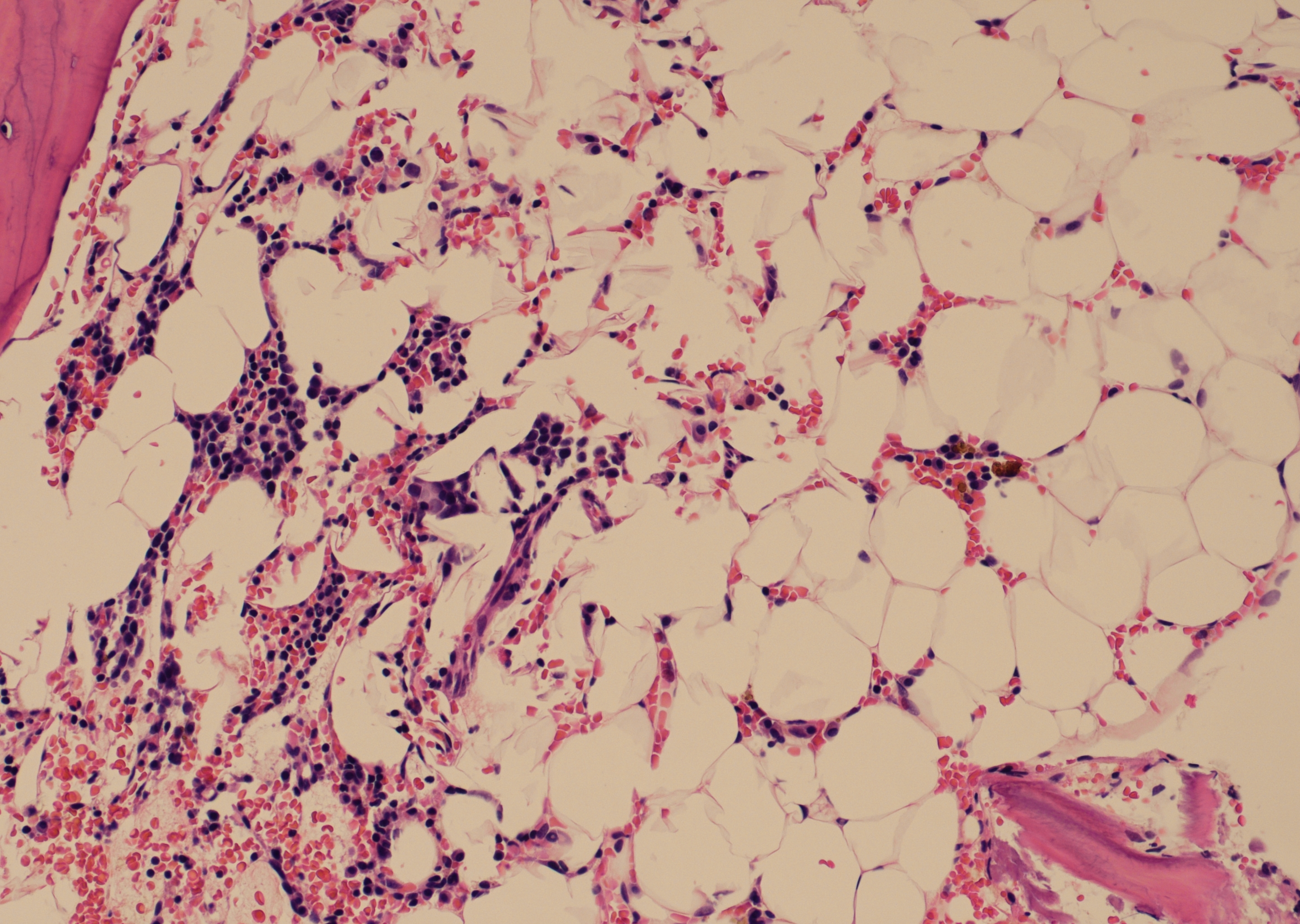

Supplement: Supplementary file 2 — Supplementary Material 2 [file 40364_2024_717_MOESM2_ESM.jpg]
