# Supplementary material for: A germline FLT3 variant in aplastic anemia
Source: Biomark Res. 2025 Jan 7;13:4. doi: 10.1186/s40364-024-00717-3 (PMC11707911; doi:10.1186/s40364-024-00717-3)
Supplement: Supplementary file 3 — Supplementary Material 3 [file 40364_2024_717_MOESM3_ESM.docx]

**On Presentation**

**Flow cytometry results**

-There are 0.04% CD34+ and/or CD117+ blasts.

-There was no significant loss of CD10 in 13.83% maturing granulocytes.

-There was no significant aberrant expression of CD56 in granulocytes or 1.54% monocytes.

-There were 9.21% B-cells.

-There were 63.62% T-cells.

-CD4/CD8 = 2.79

-There is no clonal B-cell expansion.

-There are no loss of pan T-cell markers.

| **Blast/MDS Panel** | | | | |  |
| --- | --- | --- | --- | --- | --- |
| Marker | % Gated | % Total | Gate | Intensity | |
| CD19 | 0.35 | 0.02 | Blasts | Dim | |
| CD33 | 12.28 | 0.56 | Blasts | Dim | |
| CD10 | 16.14 | 0.73 | Blasts | Dim | |
| CD34 | 1.32 | 0.06 | Blasts | Dim | |
| CD45 | 100 | 4.54 | Blasts | Dim | |
| CD117 | 1.23 | 0.06 | Blasts | Dim | |
| CD7 | 21.05 | 0.95 | Blasts | Dim | |
| CD13 | 19.65 | 0.89 | Blasts | Dim | |
| CD56 | 2.72 | 0.12 | Blasts | Moderate | |
| CD16 | 6.23 | 0.28 | Blasts | Moderate | |

|  |  |  |  |  | |
| --- | --- | --- | --- | --- | --- |
| **B Cell** | | | | |  |
| Marker | % Gated | % Total | Gate | Intensity | |
| Kappa | 49.15 | 4.53 | B Cells | Moderate | |
| Lambda | 40.21 | 3.7 | B Cells | Moderate | |
| Kappa/Lambda Ratio | 1.22 |  |  |  | |
| CD20 | 100 | 9.21 | B Cells | Dim | |
| CD23 | 85.32 | 7.86 | B Cells | Moderate | |
| CD10 | 0.4 | 0.3 | Lymphocytes | Dim | |
| CD5 | 86.89 | 65.63 | Lymphocytes | Moderate | |
| CD19 | 11.08 | 8.37 | Lymphocytes | Dim | |
| CD45 | 100 | 75.53 | Lymphocytes | Moderate | |
| CD19+5+ | 1.45 | 1.09 | Lymphocytes | Dim | |
| CD19+10+ | 0.13 | 0.1 | Lymphocytes | Dim | |
| CD38 | 2.95 | 0.27 | B Cells | Moderate | |

|  | |  | |  | |  | |  | | |
| --- | --- | --- | --- | --- | --- | --- | --- | --- | --- | --- |
| **TRBC1 PANEL** | | | | | | | | | |  |
| Marker | % Gated | | % Total | | Gate | | Intensity | |  |  |
| TRBC1 | 39.55 | | 25.16 | | T Cells | | Dim | |  |  |
| TCRrd | 0.13 | | 0.08 | | T Cells | | Dim | |  |  |
| CD4 | 71.39 | | 45.42 | | T Cells | | Moderate | |  |  |
| CD3 | 100 | | 63.62 | | T Cells | | Moderate | |  |  |
| CD5 | 98.9 | | 62.93 | | T Cells | | Moderate | |  |  |
| TCRaß | 99.6 | | 63.37 | | T Cells | | Moderate | |  |  |
| CD2 | 98.73 | | 62.82 | | T Cells | | Dim | |  |  |
| CD7 | 93.42 | | 59.44 | | T Cells | | Moderate | |  |  |
| CD8 | 25.58 | | 16.27 | | T Cells | | Moderate | |  |  |
| CD45 | 99.25 | | 63.15 | | T Cells | | Moderate | |  |  |
| CD4/CD8 Ratio | 2.79 | |  | |  | |  | |  |  |

**Bone marrow aspiration and biopsy**

The bone marrow biopsy is hypocellular (5-20%). Trilineage hematopoiesis is decreased without definite increase in blasts. The myeloid to erythroid ratio is increased. The megakaryocytes are markedly reduced in number. Most megakaryocytes are morphologically unremarkable with rare hypolobated forms. There is no increase in blasts. There are no granulomas. There is no metastatic tumor seen. The bony trabeculae are unremarkable. The bone marrow aspirate is highly hemodiluted and noncontributory.

IMMUNOHISTOCHEMISTRY:

Immunohistochemical stains are performed on paraffin embedded block B1 and are as follows:

CD3: Scattered small T-cells.

CD20: Scattered small B-cells, <5%.

CD34: Rare positive cells, <5%.

CD117: Rare positive cells, <5%. Slightly increased mast cells are present.

CD61: Highlights markedly reduced in number megakaryocytes.

Myeloperoxidase: Highlights myeloid hypoplasia.

Alpha-1 spectrin: Highlights nucleated erythroid precursors and mature red blood cells.

P53: Wild type

**One year post treatment - immunosuppressive therapy with horse anti-thymocyte globulin (ATG)/cyclosporine/eltrombopag**

**Flow cytometry**

-There are 0.22% CD34+ and/or CD117+ blasts.

-There is no significant loss of CD10 in 47.70% maturing granulocytes.

-There is no significant aberrant expression of CD56 in granulocytes or 8.95% monocytes.

-There are 2.66% B-cells.

-There are 21.18% T-cells.

-CD4/CD8 = 1.97

-There is no clonal B-cell expansion.

-There is no loss of pan T-cell markers or clonal expansion by TRBC1 expression.

| **Blast/MDS Panel** | | | | | |
| --- | --- | --- | --- | --- | --- |
| Marker | % Gated | % Total | Gate | Intensity |  |
| CD19 | 1.58 | 0.12 | Blasts | Dim |  |
| CD33 | 23.42 | 1.80 | Blasts | Dim |  |
| CD10 | 2.92 | 0.22 | Blasts | Dim |  |
| CD34 | 2.92 | 0.22 | Blasts | Negative |  |
| CD45 | 100.00 | 7.70 | Blasts | Dim |  |
| CD117 | 2.72 | 0.21 | Blasts | Dim |  |
| CD7 | 2.39 | 0.18 | Blasts | Dim |  |
| CD13 | 7.46 | 0.57 | Blasts | Dim |  |
| CD56 | 8.17 | 0.63 | Blasts | Dim |  |
| CD16 | 3.54 | 0.27 | Blasts | Dim |  |

|  | |  | |  | |  | |  | | |
| --- | --- | --- | --- | --- | --- | --- | --- | --- | --- | --- |
| **B Cell** | | | | | | | | | |  |
| Marker | % Gated | | % Total | | Gate | | Intensity | |  |  |
| Kappa | 47.35 | | 1.32 | | B Cells | | Moderate | |  |  |
| Lambda | 51.79 | | 1.45 | | B Cells | | Moderate | |  |  |
| Kappa/Lambda Ratio | 0.91 | |  | |  | |  | |  |  |
| CD20 | 95.14 | | 2.66 | | B Cells | | Dim | |  |  |
| CD23 | 75.25 | | 2.10 | | B Cells | | Dim | |  |  |
| CD10 | 0.92 | | 0.24 | | Lymphocytes | | Dim | |  |  |
| CD5 | 85.27 | | 21.77 | | Lymphocytes | | Moderate | |  |  |
| CD19 | 10.95 | | 2.80 | | Lymphocytes | | Dim | |  |  |
| CD45 | 100.00 | | 25.53 | | Lymphocytes | | Moderate | |  |  |
| CD19+5+ | 0.74 | | 0.19 | | Lymphocytes | | Dim | |  |  |
| CD19+10+ | 0.94 | | 0.24 | | Lymphocytes | | Dim | |  |  |
| CD38 | 15.88 | | 0.44 | | B Cells | | Moderate | |  |  |

|  | |  | |  | |  | |  | | |
| --- | --- | --- | --- | --- | --- | --- | --- | --- | --- | --- |
| **TRBC1 PANEL** | | | | | | | | | |  |
| Marker | % Gated | | % Total | | Gate | | Intensity | |  |  |
| TRBC1 | 36.30 | | 7.69 | | T Cells | | Dim | |  |  |
| TCRrd | 0.15 | | 0.03 | | T Cells | | Dim | |  |  |
| CD4 | 64.08 | | 13.58 | | T Cells | | Moderate | |  |  |
| CD3 | 100.00 | | 21.18 | | T Cells | | Moderate | |  |  |
| CD5 | 97.68 | | 20.69 | | T Cells | | Moderate | |  |  |
| TCRaß | 97.04 | | 20.56 | | T Cells | | Dim | |  |  |
| CD2 | 98.61 | | 20.89 | | T Cells | | Dim | |  |  |
| CD7 | 88.61 | | 18.77 | | T Cells | | Dim | |  |  |
| CD8 | 32.51 | | 6.89 | | T Cells | | Moderate | |  |  |
| CD45 | 99.26 | | 21.03 | | T Cells | | Moderate | |  |  |
| CD4/CD8 Ratio | 1.97 | |  | |  | |  | |  |  |

**Bone marrow aspiration and biopsy**

The bone marrow biopsy is variable cellular (20-50%). Trilineage hematopoiesis is present with full maturation. The myeloid to erythroid ratio is within normal limits. The megakaryocytes are adequate in number. Most megakaryocytes are morphologically unremarkable with rare hypolobated forms. There is no increase in blasts or lymphocytes. There are no granulomas. There is no metastatic tumor seen. The bony trabeculae are unremarkable. The clot contains peripheral blood only.

There is trilineage hematopoiesis with maturation. Myeloid lineage shows full maturation with no increase in blasts. Erythroid lineage shows progressive maturation. The megakaryocytes are adequate in number. Most megakaryocytes are morphologically unremarkable with rare hypolobated forms. No definite atypical lymphocytes seen. No apparent dysplasia seen. A differential count was performed on 500 cells and reported below. Stainable iron is present. No ring sideroblasts are seen.

| Aspirate smear manual counts (total number of cells) | Percentage | Normal range |
| --- | --- | --- |
| Blast % Manual | 0 | 0-3 |
| Promyelocyte % Manual | 0 | 3-12 |
| Myelocyte % Manual | 10 | 4-13 |
| Metamyelocyte % Manual | 2 | 2-6 |
| Bands/Polys % Manual | 37 | 22-46 |
| Eosinophil % Manual | 2 | 0-4 |
| Lymphocyte % Manual | 22 | 6-20 |
| Plasma Cell % Manual | 0 | 0-1 |
| Monocyte % Manual | 3 | 0-3 |
| Erythroid % Manual | 24 | 13-40 |
| Basophil % Manual | 0 | 0-1 |

IMMUNOHISTOCHEMISTRY:

Immunohistochemical stains are performed on paraffin-embedded block B1and are as follows:

CD3: Scattered small T-cells, 5-10%.

CD20: Scattered small B-cells, <5%.

CD34: Rare positive cells, <3%.

CD117: Rare positive cells, <5%. A few mast cells are present.

CD61: Highlights adequate in number megakaryocytes.

Myeloperoxidase: Highlights myeloid cells.

Alpha-1 spectrin: Highlights nucleated erythroid precursors and mature red blood cells.

M:E ratio is within normal **limits.**

P53: Wild type.
